# Estimating the frost damage index in lettuce using UAV-based RGB and multispectral images

**DOI:** 10.3389/fpls.2023.1242948

**Published:** 2024-01-04

**Authors:** Yiwen Liu, Songtao Ban, Shiwei Wei, Linyi Li, Minglu Tian, Dong Hu, Weizhen Liu, Tao Yuan

**Affiliations:** ^1^ College of Information Technology, Shanghai Ocean University, Shanghai, China; ^2^ Institute of Agricultural Science and Technology Information, Shanghai Academy of Agricultural Sciences, Shanghai, China; ^3^ Key Laboratory of Intelligent Agricultural Technology (Yangtze River Delta), Ministry of Agriculture and Rural Affairs, Shanghai, China; ^4^ Jinshan Experimental Station, Shanghai Agrobiological Gene Center, Shanghai, China; ^5^ School of Computer and Artificial Intelligence, Wuhan University of Technology, Wuhan, China

**Keywords:** lettuce, frost damage, unmanned aerial vehicle, high-throughput detection, multisource data

## Abstract

**Introduction:**

The cold stress is one of the most important factors for affecting production throughout year, so effectively evaluating frost damage is great significant to the determination of the frost tolerance in lettuce.

**Methods:**

We proposed a high-throughput method to estimate lettuce FDI based on remote sensing. Red-Green-Blue (RGB) and multispectral images of open-field lettuce suffered from frost damage were captured by Unmanned Aerial Vehicle platform. Pearson correlation analysis was employed to select FDI-sensitive features from RGB and multispectral images. Then the models were established for different FDI-sensitive features based on sensor types and different groups according to lettuce colors using multiple linear regression, support vector machine and neural network algorithms, respectively.

**Results and discussion:**

Digital number of blue and red channels, spectral reflectance at blue, red and near-infrared bands as well as six vegetation indexes (VIs) were found to be significantly related to the FDI of all lettuce groups. The high sensitivity of four modified VIs to frost damage of all lettuce groups was confirmed. The average accuracy of models were improved by 3% to 14% through a combination of multisource features. Color of lettuce had a certain impact on the monitoring of frost damage by FDI prediction models, because the accuracy of models based on green lettuce group were generally higher. The MULTISURCE-GREEN-NN model with R^2^ of 0.715 and RMSE of 0.014 had the best performance, providing a high-throughput and efficient technical tool for frost damage investigation which will assist the identification of cold-resistant green lettuce germplasm and related breeding.

## 1 Introduction

Lettuce (*Lactuca sativa* L.) is one of the most widely consumed leafy vegetables worldwide with high nutritional value(Shi et al., 2022). It is also one of the most economically important vegetable crops in the world(Soldatenko et al., 2018), with China consistently leading in production (Shatilov et al., 2019; Wang et al., 2022). Lettuces prefer cool temperatures between 7 and 24°C with an average of 18°C(Jenni, 2005), and their growth will be retarded or stagnant when temperature goes below 7°C. Frost damage of lettuce is a stress caused by low temperatures and generally occurs below 0°C(Yu and Lee, 2020). When frost damage occurs, ice nuclei are formed outside the cells and ice crystals are gradually developed as long as the low temperature continues. When the ice crystals spread into the cells, irreversible damage will occur, causing the leaves to appear watery, yellow or dark brown and the whole plant to wilt. Fresh lettuce is not storage-resistant, and its supply relies on fresh harvesting. Frost can damage the outer leaves of mature lettuce, leading to decay in handling and storage(Turini et al., 2011). As a result, low temperature is one of the most important factors threatening to supply lettuce in winter (Pirinc and Alas, 2021). Under harsh environmental conditions, crop yields can be lost ranging from 50% to 70%(Francini and Sebastiani, 2019). Due to the low availability and high market demand for lettuce in winter(Han et al., 2014), it is urgent to breed cold-resistant lettuce cultivars to keep the yield of lettuce in winter. Meanwhile, frost damage investigation is an important basement in the breeding programs of cold-resistant lettuce. The traditional method relies on manual surveys plot-by-plot in the field, which is time-consuming, laborious, subjective, and low in efficiency, especially when the number of lettuce cultivars is large. Therefore, it is of great significance to develop high-throughput methods of frost damage investigation to improve breeding efficiency.

Remote sensing based on Unmanned Aerial Vehicle (UAV), a newly developed technique for high-throughput crop growth information acquisition, has been widely used in crop monitoring under growth (Jiang et al., 2022)and various stresses such as pests, diseases, water deficit, salt-stressed(Johansen et al., 2019) and frost(Wójtowicz et al., 2016; Perry et al., 2017a; Chen et al., 2019; Choudhury et al., 2019; Goswami et al., 2019; Jełowicki et al., 2020; Millan et al., 2020; Marin et al., 2021). Although satellite remote sensing technology was also used in frost damage monitoring(Feng et al., 2009; Romanov, 2009; Romani et al., 2011; Rudorff et al., 2012; She et al., 2015; She et al., 2017; Li et al., 2021; Gabbrielli et al., 2022a; Gabbrielli et al., 2022b), the UAV-based remote sensing is more accurate in the breeding field due to its high spatial resolution. UAVs, including DJI, 3D Robotics solo and Ebee, were equipped with spectral cameras to detect frost damage of crops such as wheat(Guo et al., 2014; Wang et al., 2014; Murphy et al., 2020), maize(Choudhury et al., 2019; Goswami et al., 2019; Shu et al., 2022), oat(Macedo-Cruz et al., 2011), oilseed rape(She et al., 2015), and coffee plants(Marin et al., 2021; Marin et al., 2022). In these studies, data analysis techniques such as Pearson correlation analysis and principal component analysis were used to extract stress-related spectral features including the reflectance of different spectral bands and several commonly used vegetation indexes (VIs) such as normalized difference vegetation index (NDVI)(She et al., 2015), green NDVI, photochemical reflectance index (PRI), carotenoid reflectance index(CRI), and anthocyanin reflectance index(ARI)(Choudhury et al., 2019; Marin et al., 2021; Marin et al., 2022). Pixel-based classification with thresholds, random forest, random committee, support vector machine (SVM) and other classification methods were used to predict frost damage degree in some of these studies(Goswami et al., 2019; Jełowicki et al., 2020). Besides, regression methods such as multiple linear regression (MLR) and principal component regression were employed to predict crop yield or other physical parameters and then their changes before and after frost damage were compared to assess stress severity(Guo et al., 2014; Choudhury et al., 2019). Currently, several studies have utilized RGB and multispectral image features for lettuce, such as prediction of lettuce health(Pham et al., 2019), detection of lettuce anthocyanin content(Kim and Van Iersel, 2023) and classification of lettuce seeds(Concepcion et al., 2020). However, as far as we know, few studies have been conducted to apply these techniques to detect the frost damage of lettuce in the field during the growth stage. Unlike other crops, different varieties of lettuce in breeding trial fields exhibit significant differences in both morphology (Iceberg, Batavian, Butterhead, etc.) and color (green, red, etc.). Therefore, it remains to be seen whether these spectral features, which are widely used in crop stress monitoring, are suitable for screening the frost damage of lettuce with different varieties, and whether these non-destructive and high-throughput methods for evaluating frost damage can be applied to field investigation of lettuce breeding materials.

Frost damage to lettuce not only leads to changes of appearance, but also affects its physiological and biochemical indexes. Spectral imagers have an advantage in responding to changes in the physiological and biochemical indexes of crops due to their ability to detect reflectance spectra in the visible and near infrared wavelengths(Tao et al., 2022). This is the reason why spectral imagers were chosen in the most existing researches on frost damage of field crops(Macedo-Cruz et al., 2011; Wang et al., 2014; Yang et al., 2019; Lassalle, 2021). However, the cost of multispectral cameras is high, and the resolution of multispectral images is generally low, with insufficient texture information in the images. On the other side, Red-Green-Blue (RGB) cameras have an advantage in responding to the surface characteristics of crops due to their high spatial resolution. Despite the low cost and high resolution of RGB cameras, they cannot capture spectral information beyond the visible spectrum. The combination of data from the RGB camera and the multispectral imager allows for a comprehensive analysis of the changes in lettuce after frost damage. In early research, various methodologies utilizing data from distinct sensors were contrasted to determine the most advantageous approach (Dammer et al., 2011). Currently, there have been studies of combining multisource image features for crop monitoring (Perry et al., 2017b; Zheng et al., 2020; Li et al., 2021), confirming the improvement of detection effect when multisource image features were integrated. Thus, it is worthwhile to try to use both RGB and multispectral images to evaluate the lettuce frost damage.

Therefore, the objectives of this study were to evaluate the frost damage in lettuce by analyzing UAV-based RGB and multispectral imagery. To accomplish this objective, correlation analysis with frost damage index (FDI) was employed to find FDI-sensitive features, new FDI-sensitive VIs were constructed by modifying existing well-performing VIs and tested, and multivariate regression models using different algorithms were compared to explore the potential of lettuce FDI estimation in the field.

## 2 Materials and methods

### 2.1 Plant material and study area

**Figure 1:**
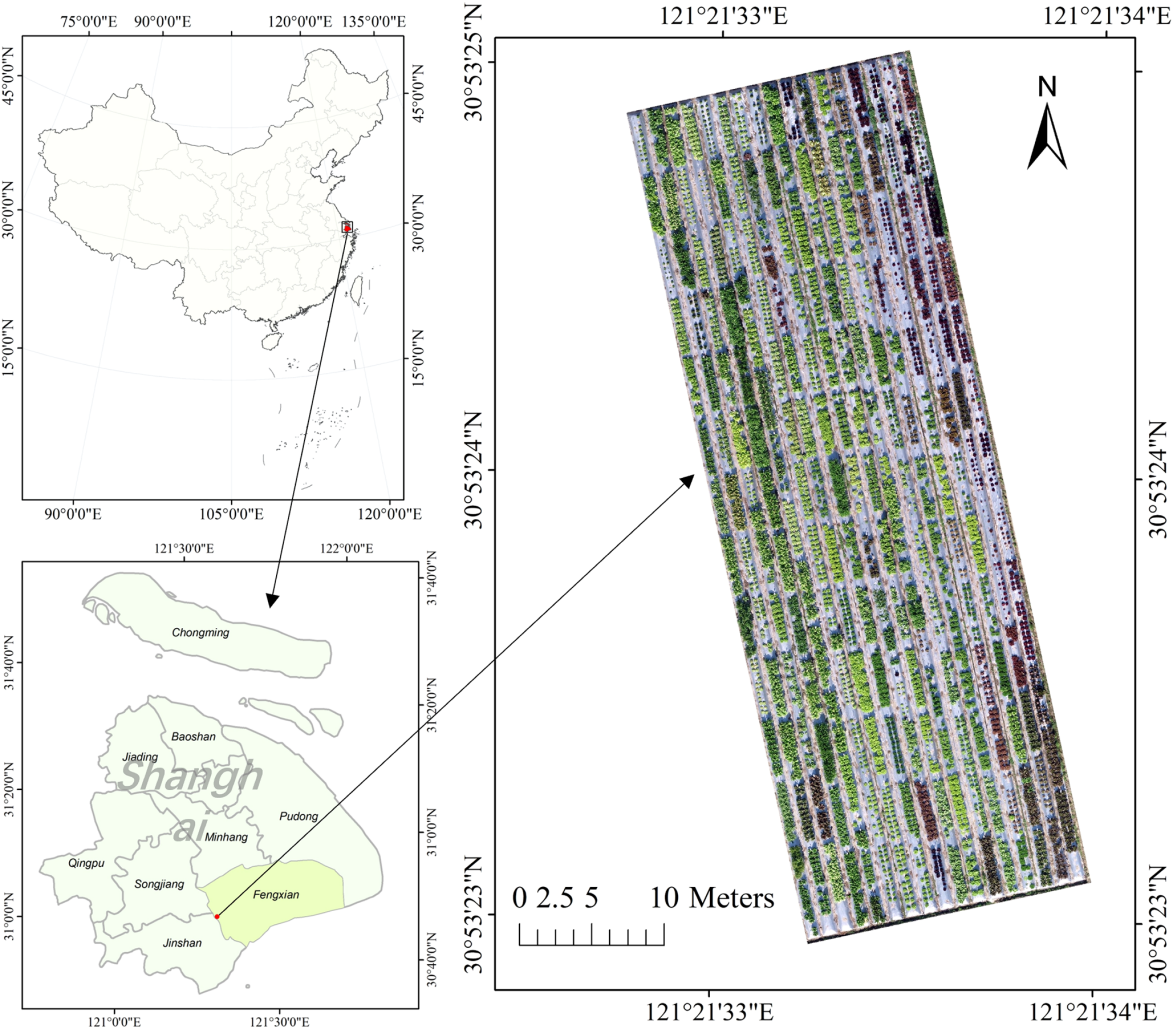
Location and RGB image of the experiment field in this study.

The experiment was performed in the test site of Shanghai Academy of Agricultural Sciences in Fengxian District, Shanghai City, China (30.891°N, 121.359°E), as shown in **Figure 1**. Fengxian District is located in the alluvial plain of the Yangtze River Delta. It has a subtropical marine monsoon climate and the average annual temperature is about 15.8°C.

A total of 209 distinct cultivars of lettuce, consisting of 160 green and 49 red varieties, were randomly assigned to 209 plots. Each plot, measuring approximately 4 m^2^ (4m x 1m), contained approximately 24 plants of each cultivar, as illustrated in **Figure 1**. All the lettuces were sown on September 24, 2020, and were transplanted to the field on October 22, 2020. The harvest period commenced around December 29, 2020.

### 2.2 Data acquisition of frost damage index

**Figure 2:**
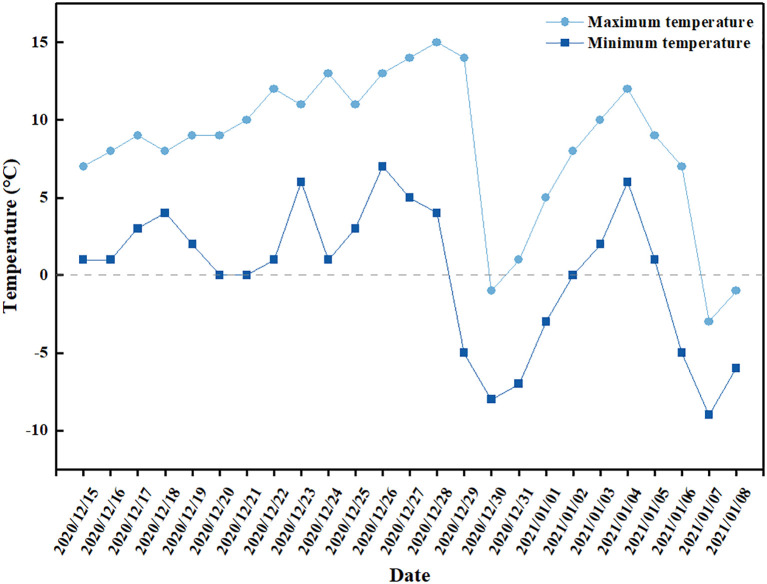
The temperature during the experiment in this study.

**Figure 3:**
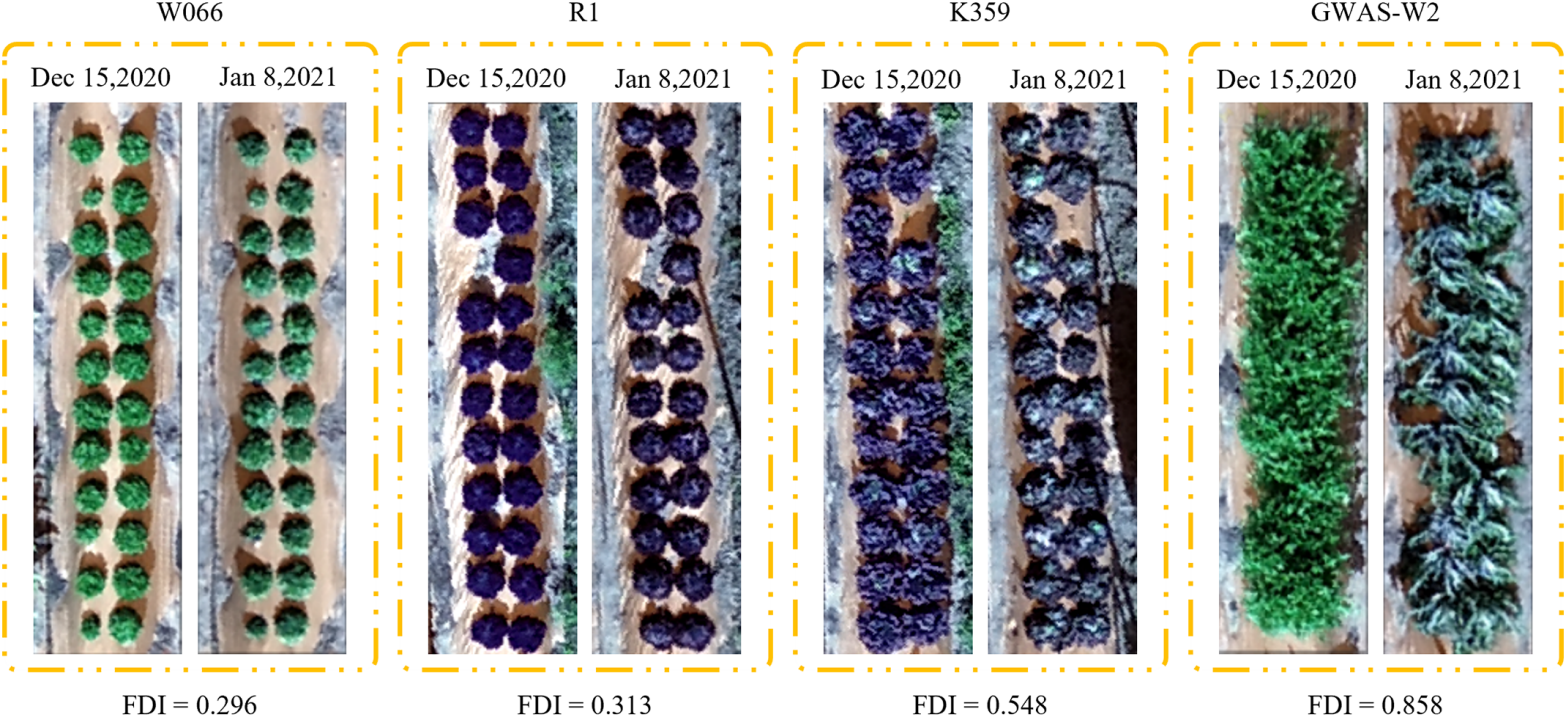
Lettuce before and lettuce after frost damage in this experiment. FDI represents frost damage index.

**Figure 2** shows the minimum and maximum temperatures from Dec 15, 2020 to Jan 8, 2021. The temperatures from Dec 15, 2020 to Dec 28, 2020 and from Jan 2, 2021 to Jan 5, 2021 range from 0 to 15°C. Lettuce leaves remain undamaged at temperatures near freezing, but are susceptible to damage at temperatures below freezing (Turini et al., 2011). The first day when temperature went below 0°C after field-planting was Dec 29, 2020, on which day the temperature dropped to -5°C. The lowest temperature, which was -9°C, appeared on Jan 7, 2021. During these two periods, from Dec 29, 2020 to Jan 1, 2021 and from Jan 6, 2021 to Jan 8, 2021, different cultivars of lettuce suffered from frost damage of different degrees, as shown in **Figure 3**.

The field investigation of FDI of each lettuce cultivar plot was carried out on Jan 8, 2021. The FDI was defined by referring to the statistical method of other damage indexes, such as chilling injury index(Fernandez-Trujillo et al., 1998; Porat et al., 2000; González-Aguilar et al., 2004; Zhao et al., 2006; Yang et al., 2011; Pan et al., 2016) and leaf disease index(Wang et al., 2015), which involved in a variety of crop stress investigations. In this research, the severity of frost damage was graded according to the characteristics of frost damage, which was shown in **Supplementary Table 1**. Then the FDI was calculated using the following equation:


$$FDI=\frac{\sum_{n}^{i}\left(PN_{i}\times i\right)}{MAX\_DL\times TPN}$$

where i is the damage level value, n is the number of damage levels counted in this plot, $PN_{i}$ is the number of plants at damage level of i in this plot, MAX_DL is the maximum damage level, TPN is the total number of plants in this plot.

### 2.3 Acquisition and processing of images

Two aerial surveys were performed on Dec 15, 2020, when the temperature had not dropped below 0°C and there was no frost damage, and Jan 8, 2021, when the lettuce had suffered from low temperature and frost damage happened. In each survey, the UAV-based RGB and multispectral images of lettuce field were acquired respectively.

The RGB images were captured by a quadrotor named DJI Phantom 4 RTK (SZ DJI Technology Co., Shenzhen, China), which is a compact and lightweight UAV with a 20-megapixel RGB camera. The flight height of flight route was set as 30 m and the corresponding ground resolution of images was 0.012 m, and the forward and side overlaps were 80% and 70%, respectively.

The multispectral images were acquired using a five-band multispectral camera with a resolution of 1280 × 980 pixels, RedEdge-M (Micasense Inc., Seattle, WA, USA), mounted on a DJI M600 Pro UAV (SZ DJI Technology Co., Shenzhen, China). The central wavelength of each band with the corresponding bandwidth was 475nm (20nm), 560nm (20nm), 668nm (10nm), 717nm (10nm), and 840nm (40nm). The flight height of flight route was set as 30 m and the corresponding ground resolution of images was 0.018 m, and the forward and side overlaps were 85% and 80%, respectively. The images of reference panel which is a gray board with 50% reflectance by the size of 15.5 cm × 15.5 cm were captured before flight missions for radiometric calibration.

The RGB and multispectral images of the whole lettuce field were generated by mosaicking the originally acquired images within the aerial survey area using Pix4Dmapper Pro (PIX4D, Lausanne, Switzerland). The multispectral images were radiometrically corrected before mosaicking according to the images of reference panel. All the mosaiced images were geometrically corrected based on the RGB image of Dec 15, 2020 using ArcGIS (ESRI, Redlands, CA, USA).

The growth of lettuces almost stopped during Dec 15, 2020 to Jan 8, 2021 and their changes were mainly caused by frost damage. On Jan 8, 2021, many lettuces had reduced their coverage and lost biometric features because of frost damage, making it difficult to separate them from the background. Therefore, we chose the image on Dec 15, 2020 to extract pure lettuce regions for image features calculation from images on Jan 8, 2021. As NDVI is one of the most sensitive indexes to vegetation cover(Liu et al., 2020; Murphy et al., 2020), decision tree classification was performed on the multispectral image (MSI) of Dec 15, 2020, when the lettuces were in health status, to separate the lettuce plants from the background by setting NDVI greater than 0.5 as the rule using ENVI (Harris Geospatial Solutions, Inc., Broomfield, CO, America). Majority/Minority analysis was then applied to the classified results to reduce small plaques. Regions of interest of lettuce plants of each plot were generated based on the classification results and converted to shapefiles which were used to extract image and spectral features from the RGB and multispectral images of Jan 8, 2021 through zonal statistics tool.

### 2.4 Image and spectral features extraction

#### 2.4.1 RGB image features

Frost damage will change the color and texture features of the RGB images of lettuce. These features will, in turn, provide information about the surface characteristics of lettuce after frost damage has occurred. The color features included the digital number (DN) of red, green, and blue channels, which were represented by R, G, and B, respectively. In addition, fifteen vegetation-related color indexes (CIs) were calculated based on DN, as listed in **Table 1**.

**Table 1:** Color indexes(CIs).

Index	Acronym	Equation	Reference
Blue Normalized Index	BNI	$\frac{B}{B+G+R}$	
Green Normalized Index	GNI	$\frac{G}{B+G+R}$	
Red Normalized Index	RNI	$\frac{R}{B+G+R}$	
Excess Green Vegetation Index	ExG	2×G−B−R	(Woebbecke et al., 1995)ADDIN
Visible Atmospherically Resistant Index	VARI	$\frac{G-R}{G+R-B}$	(Gitelson et al., 2002)
Excess Red Vegetation Index	ExR	1.4×R−G	(Meyer and Neto, 2008)
Excess Blue Vegetation Index	ExB	1.4×B−G	(Mao et al., 2003)
Excess Green minus Excess Red Vegetation Index	ExGR	ExG−ExR	(Camargo Neto, 2004)
Normalized Green-Red Difference Index	NGRDI	$\frac{G-R}{G+R}$	(Tucker, 1979)
Modified Green Red Vegetation Index	MGRVI	$\frac{G^{2}-R^{2}}{G^{2}+R^{2}}$	(Tucker, 1979)
Woebbecke Index	WI	$\frac{G-B}{R-G}$	(Woebbecke et al., 1995)
Kawashima Index	IKAW	$\frac{R-B}{R+B}$	(Kawashima and Nakatani, 1998)
Green Leaf Algorithm	GLA	$\frac{2\times G-R-B}{2\times G+R+B}$	(Louhaichi et al., 2001)
Red Green Blue Vegetation Index	RGBVI	$\frac{G^{2}-B\times R}{G^{2}+B\times R}$	(Bendig et al., 2015)
Vegetative	VEG	$\frac{G}{R^{a}B^{1-a}},\text{ a}=0.667$	(Hague et al., 2006)

For each channel of the RGB image, 8 texture features, namely, Mean (M), Variance (V), Homogeneity (H), Contrast (Con), Dissimilarity (D), Entropy (E), Second Moment (SM), and Correlation (Cor) were calculated using Gray-level Co-occurrence Matrix (GLCM). Therefore, a total of 24 texture features were calculated and named with the initials of the color channels plus the abbreviation of the texture names. The calculation formulae of texture features are as follows:


$$Mean\;\left(M\right)=\sum_{i}\sum_{j}i\times p\left(i,j\right)$$

$$Variance\;\left(V\right)=\sum_{i}\sum_{j}{\left(i-\mu \right)}^{2}p\left(i,j\right)$$

$$Homogeneity\;\left(H\right)=\sum_{i}\sum_{j}\frac{1}{1+{\left(i-j\right)}^{2}}p\left(i,j\right)$$

$$Contrast\;\left(Con\right)=\sum_{n=0}^{N_{g}}n^{2}\left\{\begin{array}{c} \sum_{i=1}^{N_{g}}\sum_{j=1}^{N_{g}}p\left(i,j\right)\\ \left|i-j\right|=n \end{array}\right\}$$

$$Dissimilarity\;\left(D\right)=\sum_{n=1}^{N_{g}-1}n\left\{\begin{array}{c} \sum_{i=1}^{N_{g}}\sum_{j=1}^{N_{g}}p\left(i,j\right)\\ \left|i-j\right|=n \end{array}\right\}$$

$$Entropy\;\left(E\right)=-\sum_{i}\sum_{j}p\left(i,j\right)\log\left(p\left(i,j\right)\right)$$

$$Second\;Moment\;\left(SM\right)=\sum_{i}\sum_{j}{\left\{p\left(i,j\right)\right\}}^{2}$$

$$Correlation\;\left(Cor\right)=\frac{\sum_{i}\sum_{j}\left(i,j\right)p\left(i,j\right)-\mu_{x}\mu_{y}}{\sigma_{x}\sigma_{y}}$$

where p(i,j) is the value of the (*i*, *j*)th entry in the gray level cooccurrence matrix; $N_{g}$ is the number of distinct gray levels in the quantized image; $\mu_{x}$ and $\sigma_{x}$ are the mean and standard deviation of x rows in matrix calculation. $\mu_{y}$ and $\sigma_{y}$ are the mean and standard deviation of y rows in the matrix calculation.

#### 2.4.2 MSI features

In addition to the changes in surface characteristics, physiological and biochemical indexes of lettuce will also undergo alterations, leading to corresponding changes in the spectral reflectance of different bands. The features extracted from MSI included the spectral reflectance of each band and different VIs. ${\text{R}}_{475}$ , ${\text{R}}_{560}$ , ${\text{R}}_{668}$ , ${\text{R}}_{717}$ and ${\text{R}}_{840}$ represented the reflectance value at specified bands. A total of twenty-three VIs were calculated based on the spectral reflectance at different bands according to the formulation in **Table 2**. Specifically, for lettuce frost damage evaluation in this study, experiential frost damage VIs (FD_VIs) were constructed by examining the correlativity between FDI and the spectral reflectance of each band, while referring to the existing FDI-sensitive VIs such as NDVI, EVI, and SIPI. These FD_VIs incorporated more bands and different constants, and their sensitivity to FDI was assessed through Pearson correlation analysis. Ultimately, four FD_VIs, namely FD_VI1, FD_VI2, FD_VI3, and FD_VI4, were established.

**Table 2:** Vegetation indexes(VIs).

Index	Acronym	Equation	Reference
Frost Damage Vegetation Index 1	FD_VI1	$\frac{{\text{R}}_{840}+{\text{R}}_{717}-{\text{R}}_{668}}{{\text{R}}_{840}+{\text{R}}_{717}+{\text{R}}_{668}}$	
Frost Damage Vegetation Index 2	FD_VI2	$\frac{{\text{R}}_{840}+{\text{R}}_{717}-{\text{R}}_{668}}{{\text{R}}_{840}+{\text{R}}_{717}+{\text{R}}_{668}-{\text{R}}_{475}}$	
Frost Damage Vegetation Index 3	FD_VI3	$\frac{{\text{R}}_{840}+{\text{R}}_{717}-{\text{R}}_{668}}{{\text{R}}_{840}+{\text{R}}_{717}+6\times {\text{R}}_{668}-7.5\times {\text{R}}_{475}+25}$	
Frost Damage Vegetation Index 4	FD_VI4	$\frac{{\text{R}}_{840}+{\text{R}}_{717}+{\text{R}}_{560}-{\text{R}}_{668}}{{\text{R}}_{840}+{\text{R}}_{717}+{\text{R}}_{668}+{\text{R}}_{560}-{\text{R}}_{475}+7}$	
Normalized Difference Vegetation Index	NDVI	$\frac{{\text{R}}_{840}-{\text{R}}_{668}}{{\text{R}}_{840}+{\text{R}}_{668}}$	(Rouse et al., 1973)
Simple Ratio Index	SR	$\frac{{\text{R}}_{840}}{{\text{R}}_{668}}$	(Pearson and Miller, 1972)
Enhanced Vegetation Index	EVI	$\frac{2.5\times \left({\text{R}}_{840}-{\text{R}}_{668}\right)}{{\text{ R}}_{840}+6\times {\text{R}}_{668}-7.5\times {\text{R}}_{475}+1}$	(Huete et al., 1999)
Atmospherically Resistant Vegetation Index	ARVI	$\frac{{\text{R}}_{840}-\left(2\times {\text{R}}_{668}-{\text{R}}_{475}\right)}{{\text{R}}_{840}+\left(2\times {\text{R}}_{668}-{\text{R}}_{475}\right)}$	(Huete et al., 1994)
Red-edge Normalized Difference Vegetation Index	RENDVI	$\frac{{\text{R}}_{840}-{\text{R}}_{717}}{{\text{R}}_{840}+{\text{R}}_{717}}$	(Fitzgerald et al., 2010)
Modified Red Edge Simple Ratio Index	mSR	$\frac{{\text{R}}_{840}-{\text{R}}_{475}}{{\text{R}}_{717}+{\text{R}}_{475}}$	(Sims and Gamon, 2002)
Modified Red Edge Normalized Difference Vegetation Index	mNDVI	$\frac{{\text{R}}_{840}-{\text{R}}_{717}}{{\text{R}}_{840}+{\text{R}}_{717}-2\times {\text{R}}_{475}}$	(Huete et al., 1997)
Red-edge Ratio Vegetation Index	RERVI	$\frac{{\text{R}}_{840}}{{\text{R}}_{717}}$	(Gitelson et al., 2005)
Photochemical Reflectance Index	PRI	$\frac{{\text{R}}_{560}-{\text{R}}_{668}}{{\text{R}}_{560}+{\text{R}}_{668}}$	(Peñuelas et al., 1995)
Structure Insensitive Pigment Index	SIPI	$\frac{{\text{R}}_{840}-{\text{R}}_{475}}{{\text{R}}_{840}+{\text{R}}_{668}}$	(Blackburn, 1998)
Red Green Ratio Index	RG	$\frac{{\text{R}}_{668}}{{\text{R}}_{560}}$	(Gamon and Surfus, 1999)
Plant Senescence Reflectance Index	PSRI	$\frac{{\text{R}}_{717}-{\text{R}}_{475}}{{\text{ R}}_{840}}$	(Merzlyak et al., 1999)
Carotenoid Reflectance Index 1	CRI1	$\frac{1\;}{{\text{R}}_{475}}-\frac{1\;}{{\text{R}}_{560}}$	(Gitelson et al., 2001b)
Carotenoid Reflectance Index 2	CRI2	$\frac{1\;}{{\text{R}}_{475}}-\frac{1\;}{{\text{R}}_{717}}$	(Gitelson et al., 2001b)
Anthocyanin Reflectance Index 1	ARI1	$\frac{1\;}{{\text{R}}_{560}}-\frac{1\;}{{\text{R}}_{717}}$	(Gitelson et al., 2001a)
Anthocyanin Reflectance Index 2	ARI2	$\left(\frac{1\;}{{\text{R}}_{560}}-\frac{1\;}{{\text{R}}_{717}}\right)\times {\text{R}}_{840}$	(Gitelson et al., 2006)
Green Normalized Difference Vegetation Index	GNDVI	$\frac{{\text{R}}_{840}-{\text{R}}_{560}}{{\text{ R}}_{840}+{\text{R}}_{560}}$	(Gitelson et al., 1996)
Green Ratio Vegetation Index	GRVI	$\frac{{\text{R}}_{840}}{{\text{R}}_{560}}$	(Gitelson et al., 2005)
Normalized Pigment/Chlorophyll Index	NPCI	$\frac{{\text{R}}_{668}-{\text{R}}_{475}}{{\text{R}}_{668}+{\text{R}}_{475}}$	(Peñuelas et al., 1994)

### 2.5 Statistical analysis and modeling algorithms

Pearson correlation analysis method was used to select the RGB and multispectral image features. The correlation analysis was employed between these features and FDI in each group. The features that reached a highly significant level (*p*<0.01) were selected. Three regression algorithms, namely MLR, SVM and neural network (NN), were used to establish the estimation models of lettuce FDI by taking selected color, texture and spectral features as independent variables.

MLR is a basic method in multiple regression analysis and widely used in remote sensing monitoring because of their good theoretical basis(Zhang et al., 2021). In this study, the MLR models were constructed based on the following calculation formula:


$$Y_{i}=\beta_{0}+\beta_{1}X_{i1}+\beta_{2}X_{i2}+\cdots +\beta_{p}X_{ip}+\epsilon_{i}$$

where $Y_{i}$ ,  p and $\epsilon_{i}$ are the FDI of the *i*th sample, the number of independent variables and the *i*th independent identically distributed normal error; $X_{ij}$ and $\beta_{j}$ are respectively the *j*th independent variable and its coefficient of the *i*th sample ( j =1, 2, …, p ).

SVM is one of the commonly used regression methods to predict physiological parameters of crops(Shah et al., 2018; Zhang et al., 2022). The essence of SVM is to construct a set of planes or hyperplanes in a high or infinite dimensional space(Cortes and Vapnik, 1995). In this study, linear kernel function and sequential minimal optimization were chosen to construct the SVM models. The value of kernel scale was 1 and the approximations of box constraint and epsilon ranged from 0.18 to 0.2 and 0.018 to 0.02, respectively.

NN is a mathematical model that simulates the brain for information processing(Agatonovic-Kustrin and Beresford, 2000). The NN models are powerful predictive tools for crop growth status(Romero et al., 2018). The network structure of NN includes the number of hidden layers, the number of nodes in each layer, the initialization of weights, the training algorithm, and the learning rate. The NN models in this study used 10 hidden layers, and the training algorithm was the Levenberg-Marquardt algorithm.

The experimental cultivars contained lettuce in both green and red colors. There may be some influence of lettuce color on the predicted results. To understand this influence, the data was divided into three groups according to the color of lettuce: a group of all the lettuce (ALL), a group of green lettuce (GREEN) and a group of red lettuce (RED). Each group was divided into a training set and a test set by stratified random sampling according to the sample ratio of 7:3. **Table 3** describes the statistical characteristics of the FDI of the samples.

**Table 3:** Descriptive statistics of the FDI of lettuce.

Statistical characteristics	ALL		GREEN		RED	
	Training set	Test set	Training set	Test set	Training set	Test set
Mean	0.525	0.53	0.518	0.517	0.562	0.541
Median	0.533	0.525	0.514	0.51	0.56	0.544
Mode	0.45	0.278	0.45	0.375	0.6	0.714
Standard deviation	0.181	0.189	0.188	0.19	0.164	0.172
Variance	0.033	0.036	0.035	0.036	0.027	0.03
Minimum value	0.2	0.2	0.2	0.214	0.286	0.29
Maximum value	1	1	1	1	0.991	0.842
Sample size	146	63	112	48	35	14

**Figure 4:**
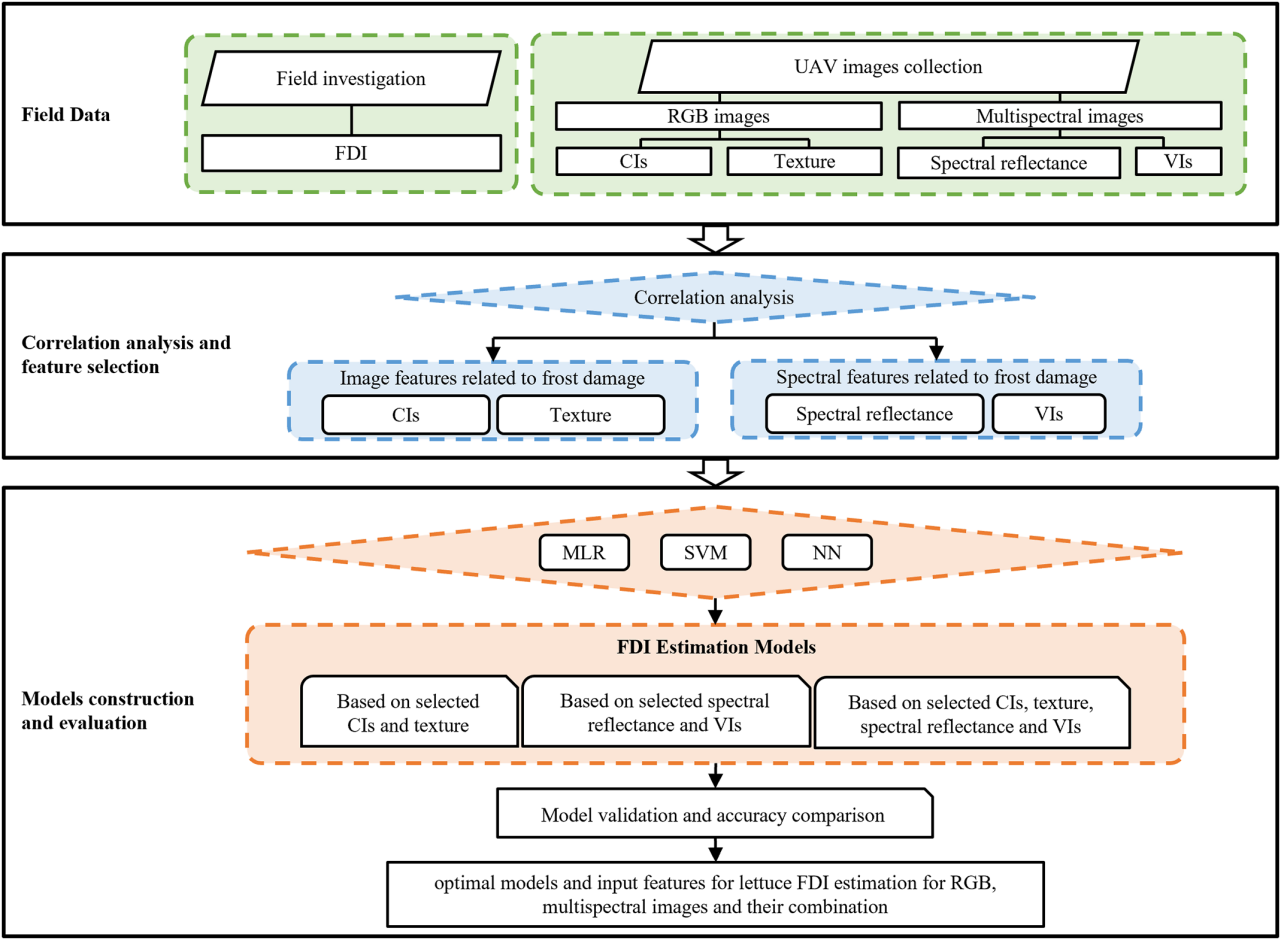
Experiment methodology and procedure of statistical analysis in this study. MLR, SVM and NN represent multiple linear regression, support vector machine and neural network, respectively.


The procedure of statistical analysis is summarized by the flowchart in **Figure 4**. Coefficient of determination (R-squared, R^2^), Root Mean Square Error (RMSE) and Mean Absolute Error (MAE) were used to evaluate the accuracy of the estimation models established with FDI as the dependent variable. The value of R^2^ (0≤R^2^ ≤ 1) determines the degree of closeness of correlation. The larger R^2^ is, the closer the relationship between dependent and independent variables is. RMSE and MAE are used to measure the deviation between the predicted and actual values. The smaller RMSE and MAE are, the closer the predicted values are to the actual values. Therefore, the closer R^2^ is to 1, RMSE is to 0 and MAE is to 0, the higher the accuracy of model will be. The formulas are as follows:

$$R^{2}=\frac{\sum_{i=1}^{n}{\left(y_{i}-{\hat{y}}_{i}\right)}^{2}}{\sum_{i=1}^{n}{\left(y_{i}-\bar{y}\right)}^{2}}$$

$$RMSE=\sqrt{\frac{\sum_{i=1}^{n}{\left(y_{i}-{\hat{y}}_{i}\right)}^{2}}{n}}$$

$$MAE=\frac{1}{n}\sum_{i=1}^{n}\left|y_{i}-{\hat{y}}_{i}\right|$$

where $y_{i}$ , ${\hat{y}}_{i}\;$ and $\bar{y}$ are the actual FDI, the predicted FDI and the average of actual FDI, respectively; n is the number of samples.

## 3 Results

### 3.1 Correlation analysis between RGB and multispectral image features and FDI

**Figure 5:**
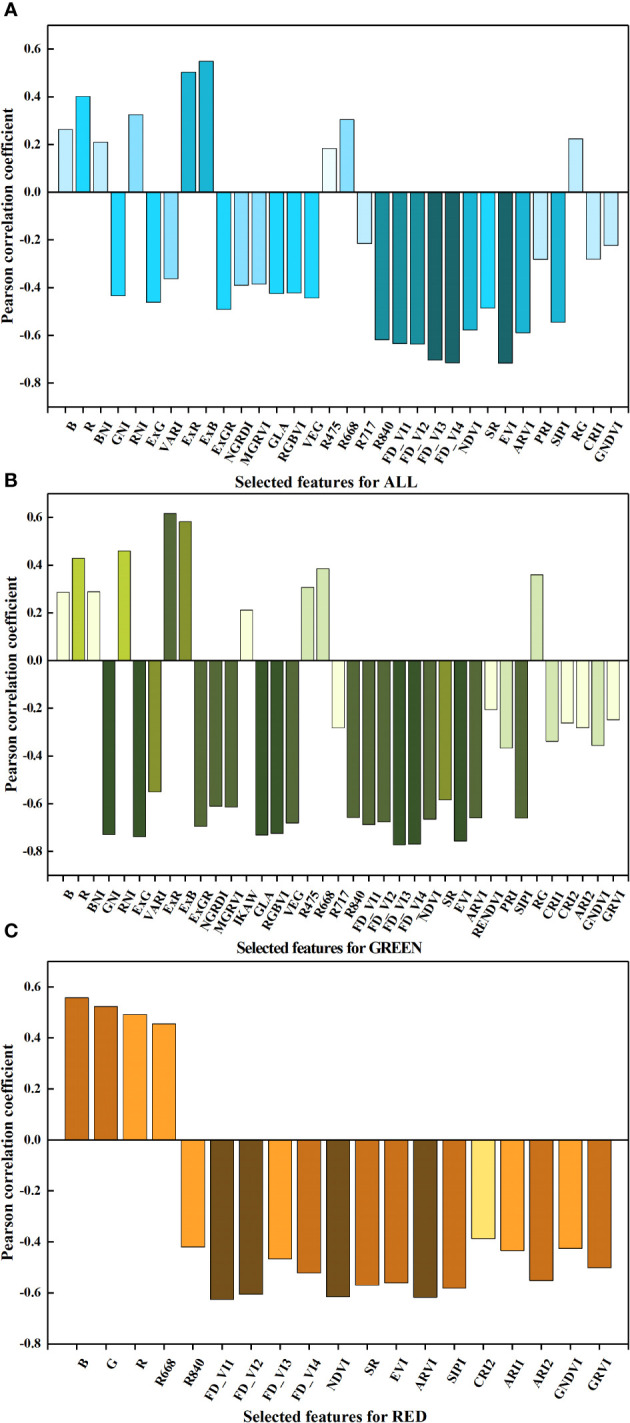
The features with significant correlation with FDI for the group of **(A)** ALL, **(B)** GREEN, and **(C)** RED.

**Supplementary Figure 1** illustrates the correlation between the RGB and multispectral image features and FDI for three distinct lettuce groups. The results reveal that certain CIs demonstrated a significant correlation with FDI, while all texture features exhibited no noticeable correlation with FDI. The majority of MSI features displayed a significant correlation with FDI, and their correlation trends with lettuce FDI remained largely consistent within each group. Notably, the correlations between certain features, primarily CIs, and FDI exhibited opposing trends for GREEN and RED. Subsequent to the correlation analysis (*p*<0.01), features with absolute Pearson correlation coefficients (absolute *r*) greater than 0.181, 0.202, and 0.358 were selected respectively for ALL, GREEN and RED for further analysis, as depicted in **Figure 5**.

For the ALL group, a total of 32 features were selected, including 15 features from the RGB images and 17 features from the multispectral images. The CIs derived from the RGB images exhibited low correlations with FDI (absolute *r*< 0.55), with ExR and ExB demonstrating absolute *r* values higher than 0.5, and the highest absolute *r* recorded at 0.549. The correlations between various MSI features and FDI displayed considerable disparity (absolute *r* arranged from 0.183 to 0.717), with ${\text{R}}_{840}$ , the four FD_VIs and EVI surpassing an absolute *r* of 0.6, and the highest absolute *r* observed for EVI.

For the group of GREEN, 16 RGB image features and 21 MSI features were selected. The absolute *r* of RGB image features were all above 0.21 (mostly surpassing 0.55) with ExG recording the highest absolute *r* of 0.738. Specifically, GNI, ExG, GLA, and RGBVI exhibited absolute *r* values exceeding 0.72. As for the MSI features, FD_VI3, FD_VI4, EVI, FD_VI1, FD_VI2, NDVI, SIPI, ARVI, and R_840 demonstrated absolute *r* values greater than 0.65. Among them, FD_VI3 attained the maximum absolute *r* of 0.773.

For the group of RED, a total of 19 features were selected, including 3 RGB image features and 16 MSI features. The selected RGB image features were B, G and R with absolute *r* of 0.558, 0.523, and 0.492, respectively. They all showed positive correlations with FDI. The MSI features with absolute *r* above 0.6 were FD_VI1, FD_VI2, ARVI and NDVI, and FD_VI1 had the highest absolute *r* of 0.626. Additionally, ${\text{R}}_{668}$ was the only feature that displayed a positive correlation with FDI.

Notably, the four newly proposed FD_VIs were explicitly correlated with FDI and displayed exceptional performance across the three groups of lettuce data, highlighting their broad utility in accurately evaluating the impact of frost damage.

### 3.2 FDI estimation models based on RGB image features

**Figure 6:**
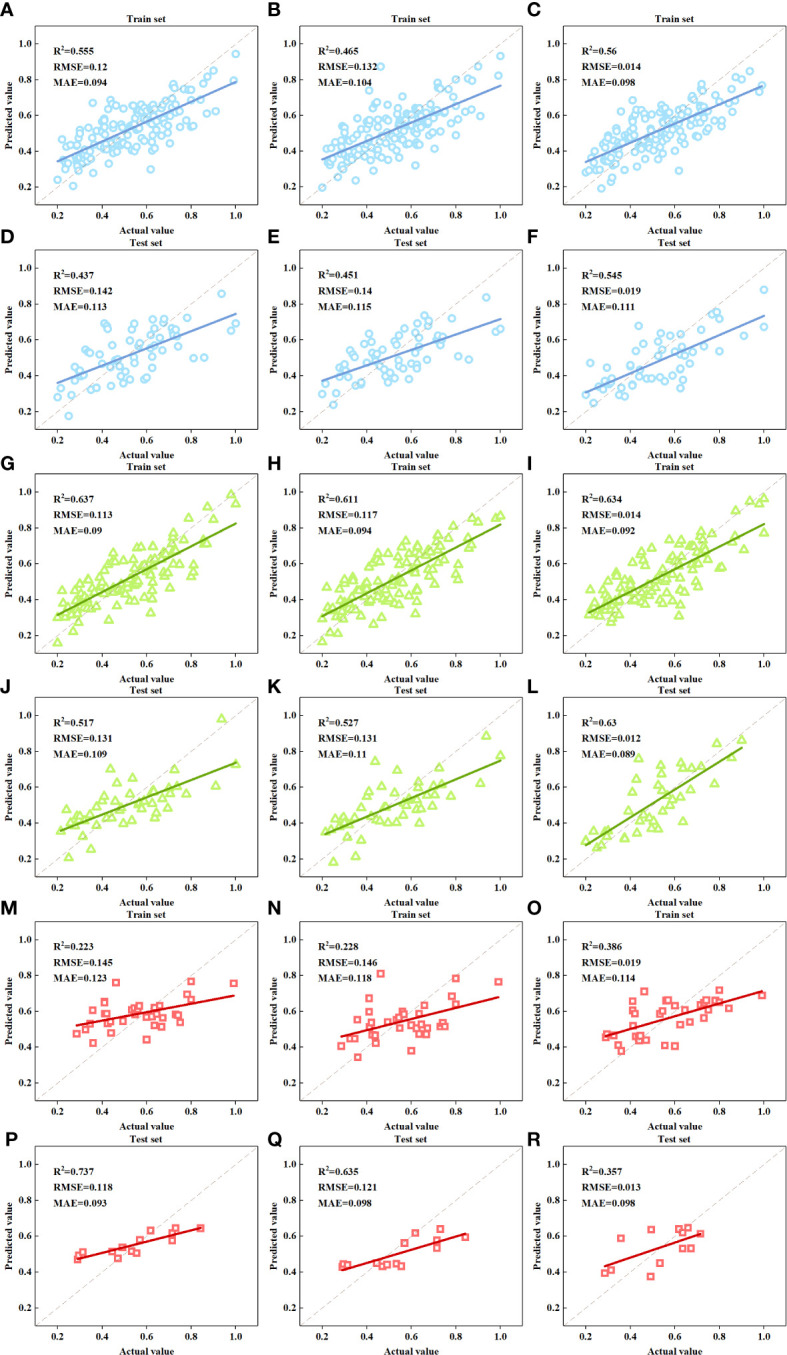
Scatter plots of the actual FDI and the predicted FDI. **(A, D).** RGB-ALL-MLR model; **(B, E).** RGB-ALL-SVM model; **(C, F).** RGB-ALL-NN model; **(G, J).** RGB-GREEN-MLR model; **(H, K).** RGB-GREEN-SVM model; **(I, L).** RGB-GREEN-NN model; **(M, P).** RGB-RED-MLR model; **(N, Q).** RGB-RED-SVM model; **(O, R).** RGB-RED-NN model.

The estimation models were constructed with selected RGB image features as independent variables and FDI as a dependent variable by using MLR, SVM and NN algorithms. The accuracy of models was demonstrated in **Table 4** and **Figure 6**. For the models of the group of ALL, the R^2^ and RMSE of training set ranged from 0.465 to 0.56 and 0.014 to 0.132 with MAE between 0.094 and 0.104, and the R^2^ and RMSE of test set ranged from 0.437 to 0.545 and 0.019 to 0.142 with MAE between 0.111 and 0.115. The FDI estimation accuracy of models for GREEN were highest among the three groups with the R^2^ of training and test sets between 0.517 and 0.637, RMSE between 0.012 and 0.131 and MAE between 0.090 and 0.094. The performance of FDI estimation models for RED was neither sufficient nor stable with R^2^ ranging from 0.223 to 0.386 for the training set, but from 0.357 to 0.737 for the test set. For all the three groups, the models using NN algorithm always achieved better accuracy and stability than others with higher R^2^, lower RMSE and lower MAE, and the difference of R^2^, RMSE and MAE between the training and test set were smaller.

**Table 4:** Accuracy of the FDI estimation models based on RGB image features.

Feature Source	Group	Model	Training set			Test set		
			R^2^	RMSE	MAE	R^2^	RMSE	MAE
**RGB**	**ALL**	**MLR**	0.555	0.120	0.094	0.437	0.142	0.113
		**SVM**	0.465	0.132	0.104	0.451	0.140	0.115
		**NN**	0.560	0.014	0.098	0.545	0.019	0.111
	**GREEN**	**MLR**	0.637	0.113	0.090	0.517	0.131	0.109
		**SVM**	0.611	0.117	0.094	0.527	0.131	0.110
		**NN**	0.634	0.014	0.092	0.630	0.012	0.089
	**RED**	**MLR**	0.223	0.145	0.123	0.737	0.118	0.093
		**SVM**	0.228	0.146	0.118	0.635	0.121	0.098
		**NN**	0.386	0.019	0.114	0.357	0.013	0.098

### 3.3 FDI estimation models based on MSI features

**Figure 7:**
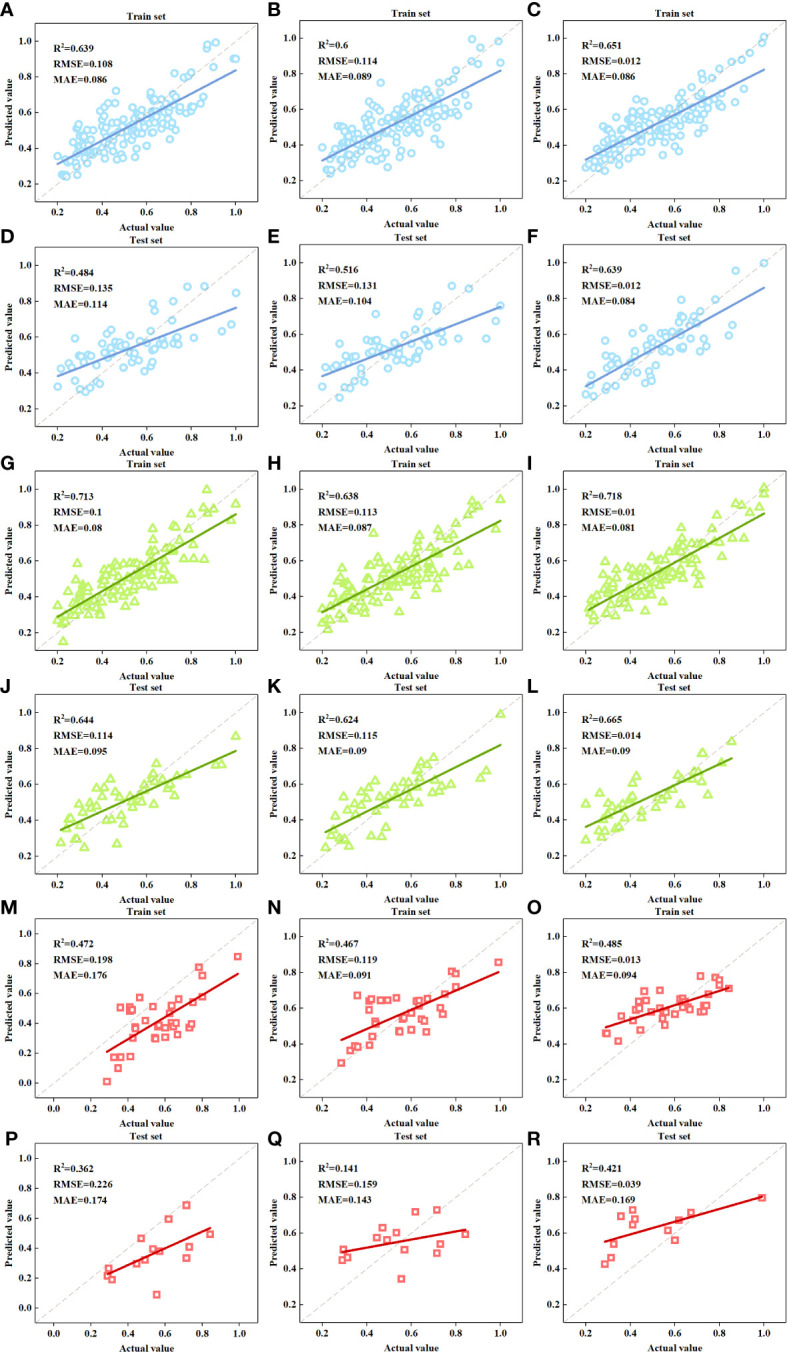
Scatter plots of the actual FDI and the predicted FDI. **(A, D).** MSI-ALL-MLR model; **(B, E).** MSI-ALL-SVM model; **(C, F).** MSI-ALL-NN model; **(G, J).** MSI-GREEN-MLR model; **(H, K).** MSI-GREEN-SVM model; **(I, L).** MSI-GREEN-NN model; **(M, P).** MSI-RED-MLR model; **(N, Q).** MSI-RED-SVM model; **(O, R).** MSI-RED-NN model.

The FDI estimation models based on selected MSI features were constructed using MLR, SVM and NN, respectively. **Table 5** and **Figure 7** illustrate the accuracy of FDI prediction. All the models based on MSI features had better performance than those based on RGB image features. The R^2^ of models by different algorithms for the group of ALL in the training set were relatively close, ranging from 0.6 to 0.651; but the R^2^ of test set varied from 0.484 to 0.639. The models for GREEN had better accuracy than other groups with R^2^ up to 0.718 and 0.665, RSME down to 0.01 and 0.014 and MAE down to 0.080 and 0.090 for the training and test set, respectively. The models for RED were improved by using MSI features, but still not good enough for FDI estimation with R^2^ lower than 0.5. As for the algorithms, NN was still superior to MLR and SVM.

**Table 5:** Accuracy of the FDI estimation models based on MSI features.

Feature Source	Group	Model	Training set			Test set		
			R^2^	RMSE	MAE	R^2^	RMSE	MAE
**MSI**	**ALL**	**MLR**	0.639	0.108	0.086	0.484	0.135	0.114
		**SVM**	0.600	0.114	0.089	0.516	0.131	0.104
		**NN**	0.651	0.012	0.086	0.639	0.012	0.084
	**GREEN**	**MLR**	0.713	0.100	0.080	0.644	0.114	0.095
		**SVM**	0.638	0.113	0.087	0.624	0.115	0.090
		**NN**	0.718	0.010	0.081	0.665	0.014	0.090
	**RED**	**MLR**	0.472	0.198	0.176	0.362	0.226	0.174
		**SVM**	0.467	0.119	0.091	0.141	0.159	0.143
		**NN**	0.485	0.013	0.094	0.421	0.039	0.169

### 3.4 FDI estimation models based on multisource features

**Figure 8:**
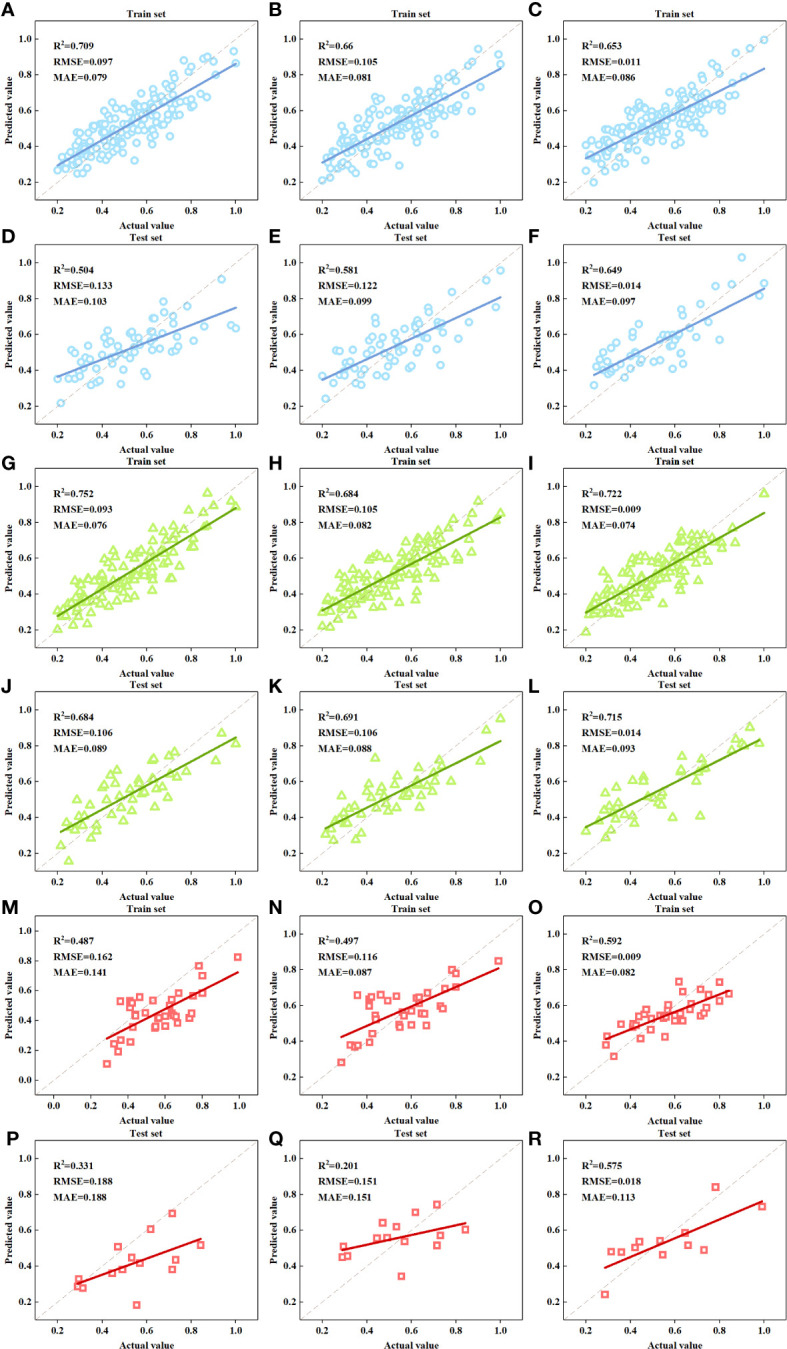
Scatter plots of the actual FDI and the predicted FDI. **(A, D).** MULTISOURCE-ALL-MLR model; **(B, E).** MULTISOURCE-ALL-SVM model; **(C, F).** MULTISOURCE-ALL-NN model; **(G, J)**. MULTISOURCE-GREEN-MLR model; **(H, K).** MULTISOURCE-GREEN-SVM model; **(I, L).** MULTISOURCE-GREEN-NN model; **(M, P).** MULTISOURCE-RED-MLR model; **(N, Q).** MULTISOURCE-RED-SVM model; **(O, R).** MULTISOURCE-RED-NN model.

In order to make full use of the information from different data sources, the estimation models were constructed using all selected features from both RGB and multispectral images, as shown in **Table 6** and **Figure 8**. Again, NN remained the best modeling algorithm for each group. In the training sets, The R^2^ of the NN models for ALL, GREEN, and RED raised to 0.653, 0.722, and 0.592; while the RMSE and MAE decreased to 0.011, 0.009, and 0.009, and to 0.086, 0.074 and 0.082, respectively. Correspondingly, The R^2^ of NN models in the test sets for each group increased to 0.694, 0.715, and 0.575, respectively; the RMSE dropped to 0.014, 0.014, and 0.018; and the MAE reduced to 0.097, 0.093 and 0.113.

**Table 6:** Accuracy of the FDI estimation models based on multisource features.

Feature Source	Group	Model	Training set			Test set		
			R^2^	RMSE	MAE	R^2^	RMSE	MAE
**MULTISOURCE** **(RGB and MSI)**	**ALL**	**MLR**	0.709	0.097	0.079	0.504	0.133	0.103
		**SVM**	0.660	0.105	0.081	0.581	0.122	0.099
		**NN**	0.653	0.011	0.086	0.649	0.014	0.097
	**GREEN**	**MLR**	0.752	0.093	0.076	0.684	0.106	0.089
		**SVM**	0.684	0.105	0.082	0.691	0.106	0.088
		**NN**	0.722	0.009	0.074	0.715	0.014	0.093
	**RED**	**MLR**	0.487	0.162	0.141	0.331	0.188	0.188
		**SVM**	0.497	0.116	0.087	0.201	0.151	0.151
		**NN**	0.592	0.009	0.082	0.575	0.018	0.113

**Table 7** illustrates the average accuracy of FDI estimation models with different sensors and groups. It can be seen that the predictive performance of models with multisource features were better than those with the single-source features. Therefore, it can be concluded that the multisource features are helpful in improving the accuracy of FDI prediction.

**Table 7:** The average accuracy of FDI estimation models with different sensors and groups.

Feature Source	Group	Training set			Test set		
		R^2^	RMSE	MAE	R^2^	RMSE	MAE
**RGB**	**ALL**	0.527	0.089	0.099	0.478	0.100	0.113
	**GREEN**	0.627	0.081	0.092	0.558	0.091	0.103
	**RED**	0.279	0.103	0.118	0.576	0.084	0.096
**MSI**	**ALL**	0.630	0.078	0.087	0.546	0.093	0.101
	**GREEN**	0.690	0.074	0.083	0.645	0.081	0.092
	**RED**	0.475	0.110	0.120	0.308	0.141	0.162
**MULTISOURCE** **(RGB and MSI)**	**ALL**	0.674	0.071	0.082	0.578	0.089	0.100
	**GREEN**	0.719	0.069	0.078	0.696	0.075	0.090
	**RED**	0.525	0.096	0.103	0.369	0.119	0.151

## 4 Discussion

### 4.1 Response of RGB and multispectral features to FDI of lettuce with different colors

The damage caused by frost on lettuce will change the way how solar radiation interacts with lettuce leaf cells. When frost damage happened, chlorophyll broke down and Photosynthesis was weakened(Choudhury et al., 2019), which would cause the decrease of absorption of visible light, especially the blue and red light which were mainly used for photosynthesis(Gates et al., 1965). In consequence, the reflectance of red and blue bands increased with the FDI. As can be seen in **Supplementary Figure 1B** and R of RGB image as well as ${\text{R}}_{475}$ and ${\text{R}}_{668}$ of MSI were positively correlated with FDI. For the same reason, most of the VIs (including NDVI, SR, EVI, ARVI, and SIPI) that had been proven to be related to chlorophyll content on(Susantoro et al., 2017; Chen et al., 2021; Shu et al., 2022) had significant negative correlation with FDI.

The response of green channel of RGB image (G) and reflectance at green band of MSI ( ${\text{R}}_{560}$ ) responded quite differently for green and red lettuce. The absolute r of G and ${\text{R}}_{560}$ of red lettuce were much higher than that of green one. The possible reason is that red lettuce contained much more anthocyanin which absorbed the green light(Yang et al., 2016); when anthocyanins of lettuce decomposed due to frost damage, the absorption of green light was decreased and the reflection was enhanced; therefore, the positive relations between FDI and G and ${\text{R}}_{560}$ of red lettuce were more significant. For the same reason, the anthocyanin reflectance indexes of MSI, ARI1 and ARI2, which reflected anthocyanin content, was more sensitive to FDI of red lettuce. For green lettuce, the change of color from green to yellow caused by frost damage was obvious. Although G and ${\text{R}}_{560}$ did not achieve a significant correlation with FDI of the green lettuce, this change could still make the CIs(including GNI, ExG, VARI, ExGR, NGRDI, MGRVI, GLA, RGBVI, and VEG) which enhanced the green component show much closer relation to FDI of the green lettuce than that of the red one.

The destruction of cell structure due to frost damage would cause the decrease of reflectance at NIR band(Gates et al., 1965). As a result, ${\text{R}}_{840}$ was obviously negative correlation with FDI of all lettuces. The result is similar to that obtained in an experiment evaluating maize frost damage, where it was found that the frost damage caused a sharp decline of reflectance between 720 and 1350 nm(Choudhury et al., 2019).

Although Pearson correlation analysis showed that ${\text{R}}_{717}$ and ${\text{R}}_{560}$ were weakly correlated with FDI, the four proposed FD_VIs also showed high correlations with FDI. These improvements were mainly due to the involvement of red edge band (717nm), green band (560nm) and blue band (475nm), on the basis of the near-infrared band (840nm) and red band (668nm). In particular, the correlations between FD_VI4 of all five bands and FDI of green and red lettuce reached the maximum. The possible reason is that canopy spectrum is the result of comprehensive influence of multiple factors such as internal components of leaves and canopy structure, and there are synergistic changes between FDI and the spectrum of these bands.

The correlations between the texture features and the FDI were not significant. One possible reason is that there were many cultivars with different structures in this experiment, and the difference in texture features among cultivars exceeds the difference in texture caused by frost damage.

### 4.2 Effect of different sensors and algorithms on FDI estimation models of lettuce with different colors

The lettuce FDI estimation models based on MSI features had better performance than those based on RGB image features, which is consistent with previous studies on the vegetation coverage monitoring and nitrogen accumulation estimation of rice (Zheng et al., 2018; Furukawa et al., 2021). It was because that multispectral imager typically captured more information than RGB cameras(Shu et al., 2022). While RGB cameras capture only three color channels, multispectral imagers capture more pronounced changes in reflectance at multiple bands across the electromagnetic spectrum, including near-infrared bands. This allows for the calculation of vegetation indexes that are highly sensitive to changes in plant health and vigor. This additional information can be leveraged to better detect and quantify frost damage. Some relevant studies have proved that RGB images are generally used to monitor the early growth of crops, and the information about near-infrared band provided by multispectral images is more suitable for the later growth of crops(Marcial-Pablo et al., 2019). In addition, the use of multispectral images in some studies enhanced the monitoring of biodiversity(Tait et al., 2019; Wolff et al., 2023). In this research, the monitored field lettuces were already in the harvesting period, and the changes were no longer obvious as in the early growth stage. As a result, the changes captured by the RGB camera were limited. At the same time, many cultivars of lettuce were selected in this study, the diversity of which was suitable for monitoring with a multispectral camera. However, although multispectral imagers had the advantage of high accuracy, RGB cameras could also be an alternative selection for low-cost monitoring of frost damage, for green lettuce at least. The FDI predictive accuracy was further improved by taking the combination of both RGB and multispectral image features as model independent variables, in which way all the information related to frost damage was fully used and the problem of spectrum saturation can be solved(Zhou et al., 2021; Shu et al., 2022). The conclusion that multisource data fusion can improve model accuracy has also been demonstrated in previous studies on crop monitoring(Jiang et al., 2019; Liu et al., 2019; Zhu et al., 2021).

Compared to the models trained based on RED and ALL, the models trained based on GREEN worked better. The major reason for the low accuracy of RED models was that there were only 49 red lettuce plots, and the sample size of red lettuce was too small which led to severe overfitting in most models for RED. In the future studies, the number of red lettuce cultivars should be increased in the selection of experimental cultivars to improve the sample size of the red lettuce dataset. Since green and red lettuces had different levels of secondary metabolites, they presented different colors and produced different responses after frost damage, as manifested in section 3.1. Therefore, the accuracy of models for ALL were not as good as those for GREEN. But even in the best FDI estimation model for GREEN, the RGB and multispectral image features could explain only about 70% of the variation. The differences between lettuces were not only caused by the different frost damage degrees, but also influenced by the different plant morphologies and green levels among different lettuce cultivars. The accuracy of models built by different regression algorithms did not show significant distinction for ALL and GREEN. NN models tended to be more stable than MLR and SVM with less difference of R^2^ between training and test set.

## 5 Conclusions

This study showed the feasibility of using features derived from RGB and multispectral images collected by a UAV to estimate lettuce FDI. Especially, the accuracy of models with multisource features were higher than those with single-source features. Notably, the four newly proposed FD_VIs had certain universal correlation with frost damage of lettuce with different colors. The findings could be applied for the prediction and evaluation lettuce resources tolerant to freeze by non-destructive, accurate, and high-throughput identification, providing genetic resources and theoretical basis for the cultivation and genetic improvement of new varieties of lettuce resistant to frost damage in the future. In subsequent works, the impact of differences among cultivars of lettuce on the evaluation of frost damage should be considered, and the sample size of red lettuce should be increased, so as to improve the evaluation models and improve the accuracy of evaluation results.

## Data availability statement

Data, models, or codes generated or used in the course of the study are available on GitHub at https://github.com/kwcnmm/predict-FDI.

## Author contributions

YL implemented this research and wrote the draft of the manuscript. SB and MT helped formulate research ideas and highlighted research objectives. SW provided research materials and field data. DH and TY provided guidance for technical issues. WL and LL provided constructive suggestions to clarify the expression of ideas. All authors contributed to the article and approved the submitted version.

## Supplementary material

Supplementary Figure 1 Pearson correlation analysis between RGB and multispectral image features and FDI.

Supplementary Table 1 Damage level and description for assessing frost damage of lettuce.
