# Supplementary material for: Estimating the frost damage index in lettuce using UAV-based RGB and multispectral images
Source: Front Plant Sci. 2024 Jan 4;14:1242948. doi: 10.3389/fpls.2023.1242948 (PMC10794741; doi:10.3389/fpls.2023.1242948)
Supplement: Supplementary Table 1 — Damage level and description for assessing frost damage of lettuce. [file Table_1.docx]

Supplementary Material

Article Title

Yiwen Liu, Songtao Ban, Shiwei Wei, Linyi Li, Minglu Tian*, Dong Hu, Weizhen Liu, Tao Yuan

*** Correspondence:** Minglu Tian: tianminglu@saas.sh.cn

# Supplementary Figures and Tables

## Supplementary Tables

**Supplementary Table 1.** Damage level and description for assessing frost damage of lettuce.

| **Damage Level Value** | **Description** |
| --- | --- |
| 1 | The leaves are normal and almost free from frost damage |
| 2 | Less than 1/3 of the area with brown stain caused by frost damage (mild frost damage) |
| 3 | Frost damage to 1/2 leaf area (moderate frost damage) |
| 4 | Frost damage to 2/3 leaf area (moderate to severe frost damage) |
| 5 | All leaves showed brown stain, and the plant basically died (severe frost damage) |
